# Explaining care need assessment surveys: qualitative and quantitative evaluation of state-of-the-art local and global explainable artificial intelligence methods

**DOI:** 10.1093/jamiaopen/ooaf064

**Published:** 2025-07-29

**Authors:** Necip Oğuz Şerbetci, Stefan Blüher, Paul Gellert, Ulf Leser

**Affiliations:** Institute for Computer Science, Humboldt-Universität zu Berlin, Berlin 10099, Germany; Institute of Medical Sociology and Rehabilitation Science, Charité, Berlin 10117, Germany; Institute of Medical Sociology and Rehabilitation Science, Charité, Berlin 10117, Germany; Institute for Computer Science, Humboldt-Universität zu Berlin, Berlin 10099, Germany

**Keywords:** long-term care, long-term care insurance, explainable AI, information extraction, text classification

## Abstract

**Objective:**

With extended life expectancy, the number of people in need of care has been growing. To optimally support them, it is important to know the patterns and conditions of their daily life that influence the need for support, and thus, the classification of the care need. In this study, we aim to utilize a large corpus consisting of care benefits applications to do an explorative analysis of factors affecting care need to support the tedious work of experts gathering reliable criteria for a care need assessment.

**Materials and Methods:**

We compare state-of-the-art methods from explainable artificial intelligence (XAI) as means to extract such patterns from over 72 000 German care benefits applications. We train transformer models to predict assessment results as decided by a Medical Service Unit from accompanying text notes. To understand the key factors for care need assessment and its constituent modules (such as mobility and self-therapy), we apply feature attribution methods to extract the key phrases for each prediction. These local explanations are then aggregated into global insights to derive key phrases for different modules and severity of care need over the dataset.

**Results:**

Our experiments show that transformers-based models perform slightly better than traditional bag-of-words baselines in predicting care need. We find that the bag-of-words baseline also provides useful care-relevant phrases, whereas phrases obtained through transformer explanations better balance rare and common phrases, such as diagnoses mentioned only once, and are better in assigning the correct assessment module.

**Discussion:**

Even though XAI results can become unwieldy, they let us get an understanding of thousands of documents with no extra annotations other than existing assessment outcomes.

**Conclusion:**

This work provides a systematic application and comparison of both traditional and state-of-the-art deep learning based XAI approaches to extract insights from a large corpus of text. Both traditional and deep learning approaches provide useful phrases, and we recommend using both to explore and understand large text corpora better. We will make our code available at https://github.com/oguzserbetci/explainer.

## Introduction

Rising life expectancy has increased the demand for care services, with Germany projecting 5 million care recipients in 2023 and 7.6 million by 2055.^1^ The Medical Service (Medizinischer Dienst, MD) in Germany assesses and allocates care benefits using a standardized survey instrument to ensure equitable distribution. The instrument comprises multiple choice questions and accompanying unstructured text notes justifying and detailing the care need of the assessed individual. The potential for extracting insights from these unstructured texts still remains largely untapped, which may reveal key factors influencing care need assessment, inform the development of preventive strategies, and guide personalized rehabilitative measures. Clearly, such analysis is impossible to do it by hand given the large number of assessed applications.

Natural language processing (NLP) is the computational processing of text and enables analysis of large corpora.^2^ Previous work applied a vocabulary-based analysis to probe the importance of a priori selected features.^3^ While valuable in probing existing hypotheses, this approach is limited in its ability to conduct open-ended data exploration. In contrast, we aim to uncover patterns beyond predefined categories or features. Recent transformers-based language models like BERT^4^ have shown superior performance in text classification^5^ and information extraction.^6^ However, information extraction requires annotated data for supervised training, which is costly to obtain and does not readily exist for care-relevant information extraction tasks.

To address these challenges and to facilitate open exploration of the data with transformers, we propose leveraging methods from explainable artificial intelligence (XAI). Many traditional machine learning (ML) models, such as linear bag-of-words models and decision trees, are readily interpretable.^7^ Transformers, despite its widespread use and effectiveness, are often considered “black boxes” due to the complexity of their internal mechanisms and the difficulty in interpreting their predictions.^8^ Explainable artificial intelligence methods aim to make ML predictions understandable by humans^9^ by revealing relationships between inputs, the learned model, and its outputs, while implicitly reflecting the training data the model has encountered.^10^ Previous work used XAI to extract insights in domains such as digital humanities^10^ and biology^11^ but their effectiveness in analyzing care need data is unknown.

With the aim of obtaining dataset level insights from the unstructured assessment notes, we train classifiers to predict different outcomes of the care need assessment corresponding to different areas of life from the accompanying text notes. Our study is explorative and evaluates the usage of XAI to find concrete circumstances of patients that hint toward different care. By explaining assessment outcome predictions with feature attribution methods, which put a score on each individual unit of the input—in this case subword tokens—and aggregating them, we identify phrases associated with these outcomes.

Our study is explorative and evaluates the usage of XAI to find concrete circumstances of patients that hint toward different care. These circumstances currently are encoded in manually defined lists of criteria used by the MD in their evaluations, but the quality and completeness of these lists remains unclear. We explore whether XAI technologies can help to improve these lists by learning them directly from reports in the archive instead of relying on manual curation.

## Methods

**Figure 1.**
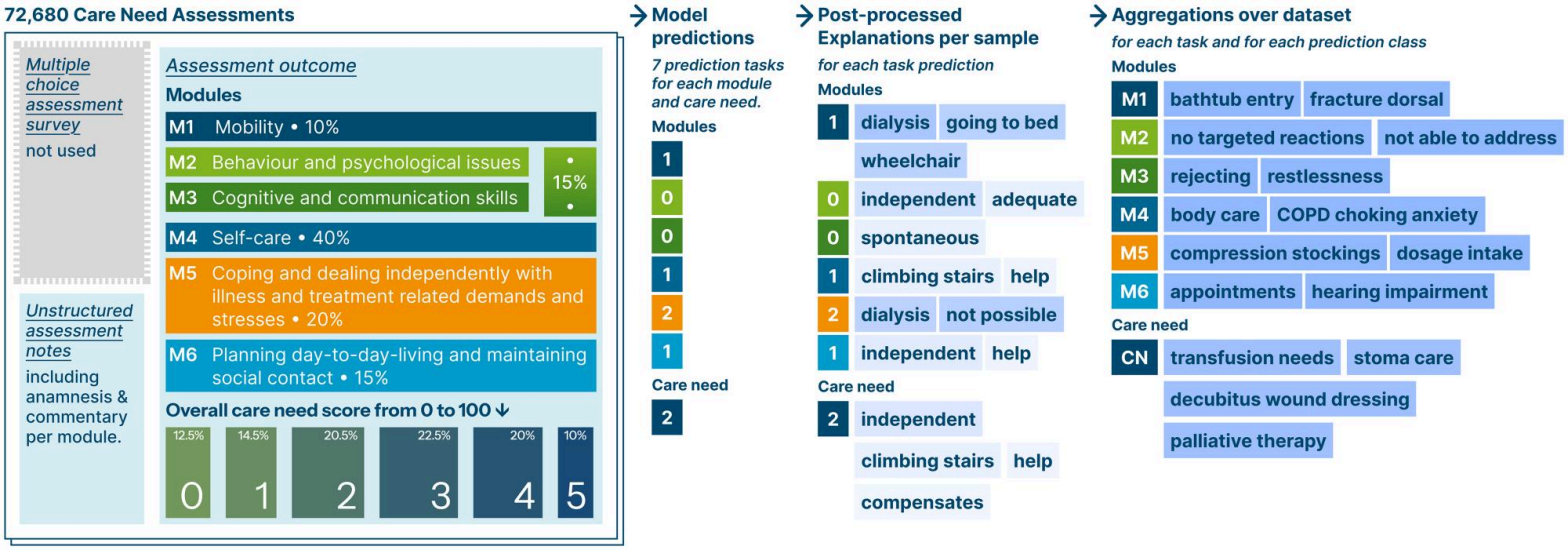
Data structure and the processing to extract important phrases from a corpus of care need assessments. Each module is scored by a multi-choice survey, from which we only use the final categorical level between 0 and 4. The module scores are also summed to the final care need score using different weights and is represented with a level 0-5. We train classifiers for module and care need levels on the assessment texts and explain each classification along each prediction task and predicted class to then aggregate these over the dataset.

This section outlines our methodological approach. First, we detail the structure of the care need assessment data, including its organization into modules, and how we process it into a dataset. Next, we detail the development of machine learning models to predict outcomes for each of the assessment modules and how we explain their predictions with XAI for the goal of extracting insights from the large assessment text notes. Finally, we explain how we aggregate explanations to obtain insights specific to each assessment module over the full dataset. Figure 1 presents both the data structure and our workflow to obtain insights.

### Care need assessment dataset

The dataset consists of 72 680 applications for long-term care benefits submitted to the Medical Service Berlin-Brandenburg, Germany (Medizinischer Dienst Berlin-Brandenburg, MD) between 2017 and 2019 obtained through the project “Health trajectories in old age paths to the need for care” (Gesundheitsverläufe im Alter—Wege in die Pflegebedürftigkeit funded by the GKV Spitzenverband). MD’s assessment instrument includes socio-demographic data, care-relevant history, medical report, current care situation, and unstructured text about living situation, household composition, and support potential by partners or other relatives (MDS, 2019). For our analysis, MD anonymized the text by hand. The assessment instrument is separated into 6 areas of daily living in corresponding assessment modules. Their weighted aggregation makes up the final score for care needs, as visualized in Figure 1. In this procedure, it is not the need for assistance that is assessed, but the applicant’s remaining independence in different areas. Based on the score determined in the assessment interview—usually by a care professional—the recommendation for classification into a specific care degree is ultimately made or no need for care is determined.

We treat each care need assessment as a single sample, aggregating assessment notes into a single unstructured text, while excluding outcome-related notes. This results in input with an average length of 1.125 tokens. The individual module levels (0-4) and the final level of care need (0-5) form the prediction targets. We divide the dataset, comprising 72 680 samples, into training, validation, and test sets containing 50 876, 7268, and 14 536 samples, respectively.

### Training predictive models on care need assessments and explaining predictions with XAI

We chose a bag-of-words ridge classifier with TF-IDF features as a baseline because it is a strong and readily explainable model for text classification.^12^ We extract 1 to 3-grams, with a minimum document frequency of 2 and maximum document frequency of N ∗ 0.3, where N is the number of training samples.

We obtain the importance of each feature by multiplying model’s coefficients with the input representation, as feature contribution depends on the input distribution. To identify phrases that are most important across the dataset and the tasks, we try 2 approaches: First, a naive approach that multiplies the coefficients with the average input representation of the training data, which is an established approach to derive feature importance from a sparse text-classifier. (Classification of text documents using sparse features: https://scikit-learn.org/stable/auto_examples/text/plot_document_classification_20newsgroups.html). Second, we propose multiplying input representation of each sample with the coefficients to obtain feature attributions, and later aggregate the sample-specific scores. By computing the feature attributions separately for each sample, we aim to exploit the variability of the feature contribution in the aggregation step.

Deep learning has proven its effectiveness in text classification by fine-tuning pretrained transformers models^5^ on supervised datasets with text and label pairs. Transformers models performance is mostly attributed to the attention mechanism, which allows each token in a sequence to dynamically focus on and weigh the relevance of all other tokens when creating its contextual representation.^13^ Pre-training and tokenization have a large impact on the performance of the learned text classifier, making it important to choose a model that is appropriate for the domain. The text we are dealing with is in German and related to clinical domain. Therefore, we choose to utilize the pre-trained weights from the German clinical language model medbert.de.^14^ This is a transformer model based on the BERT architecture that has shown state-of-the-art performance when fine-tuned in down-stream tasks.

Most BERT models are limited to 512 sub-tokens, while our samples average 1500. Rather than using long-input models like LongFormer,^15^ we follow Sun et al.^16^ We split assessments into overlapping 512-token segments, process each through BERT, then combine their representations via pooling before classification. We optimize hyperparameters using development data based on care need F1 macro scores. In these experiments, we find that best pooling over the input segments was to use average and maximum pooling followed by a layer norm.

After training Transformer models, we compare different XAI methods to obtain feature attributions for individual predictions regarding their faithfulness. One way to evaluate faithfulness is to remove tokens one-by-one and run predictions again and track the deterioration in predictive performance per number of tokens removed.^17^ The sharper and deeper the deterioration, the more faithful the feature attribution as it identified parts of the input that are most important for the model’s prediction. Specifically, we evaluate the following feature attribution approaches:

- **Attention-based explanation** utilizes the model’s learned attention weights to create feature importance mappings for input. By aggregating attention scores over the network, we obtain a heatmap that visualize how different parts of the input contribute to the model’s decision. However, the effect on different tasks and prediction classes cannot be readily distinguished.^8^
- **Gradient X Activation (LGXA)** computes feature importance by multiplying the neuron activations and output gradients throughout the network. Starting from the input layer, it traces how each neuron’s activation contributes to the final prediction while considering the gradient of the output with respect to that neuron. This method effectively captures both the forward propagation of information (through activations) and backward propagation of importance (through gradients), providing a comprehensive view of feature contributions across all network layers.^18^
- **Integrated gradients (IG)** addresses the limitations of simple gradient methods by considering the entire path from a neutral baseline (typically zero embeddings or padding tokens) to the actual input. Rather than using only the gradient at the given input as LGXA, IG averages gradients by computing the integral of the gradients along that path, multiplied by the difference between the actual activation and the baseline. This approach provides more stable and theoretically grounded attributions compared to single-gradient methods, as it satisfies desirable properties like completeness (attributions sum to the difference between output and baseline) and implementation invariance (attributions are identical for functionally equivalent networks).^19^
- **Layer-wise relevance propagation (LRP)** implements a conservative relevance flow principle, where the network’s prediction score is redistributed backwards through the layers, preserving the total relevance at each step. Using the analogy of water flowing through pipes, relevance flows from higher layers to lower layers according to the strength of neural connections with specialized propagation rules adapted for transformer architectures. This method ensures that the sum of relevance scores remains constant across layers while capturing complex interactions between features, making it particularly effective for deep neural networks with non-linear components.^20^

### Obtaining dataset level insights through XAI aggregation

The baseline bag-of-words classifier uses features of 1-3-g that exclude stop words. In contrast, the transformer feature attributions are on subtoken level. In post-processing, we merge the subtokens to form single tokens for each word with an attribution that is the sum of its subtoken. Next, we compute the 1-3 *n*-g excluding stop words and averaging the attributions. Average allows, in comparison to maximum, relevant 1-gram to overpower its 2 or 3-g. We call the spans of texts with an associated feature attribution score a phrase.

After obtaining phrases for each individual prediction, called local explanations, we proceed to derive global task-specific explanations by aggregating them. To this end, van der Linden et al.^21^ suggest a global average task importance and a novel global homogeneity-weighted task importance using entropy to identify phrases that are selectively important for a prediction class. We adapt this approach for our multi-class and multitask setting by either including both in the aggregation or discarding the class-level information. The rationale is to get insights on different tasks corresponding to different modules of the assessment, that is, different areas of life that affect care need. We discard negative attributions as their interpretation is ambiguous, for example, a negative attribution for one task might mean positive attribution for another.

Given positive attribution scores $W_{\mathrm{ij}}^{t}$^+^ for sample *i*, phrase *j*, and task *t*, the global average task importance *I*^AVG^ is defined as


$$I_{\mathrm{tj}}^{\text{AVG}}=\frac{\sum_{i}W_{ij}^{t+}}{\sum_{i:W_{\mathrm{ij}}^{t+}\neq 0}1}.$$

Similarly, the global homogeneity-weighted task importance *I*^H^ for feature *j* and task *t* is formulated as


$$I_{tj}^{\text{ATTR}}=\sqrt{\sum_{i}W_{ij}^{t+}},\;I_{tj}^{H}=(1-\frac{H_{j}-H_{\min}}{H_{\max}-H_{\min}})I_{tj}^{\text{ATTR}},\;$$

where *H_j_* is the Shannon entropy of the distribution *p_tj_*, which is the distribution of phrase *j’*s importance over all tasks and obtained through


$$p_{tj}=\frac{\sqrt{\sum_{i}W_{\mathrm{ij}}^{t+}}}{\sum_{t}\sqrt{\sum_{i}W_{ij}^{t+}}},\;H_{j}=-\sum_{t}p_{tj}\;\text{log}\;(p_{tj}),$$

and *H_min_* and *H_max_* are minimum and maximum entropy measured across all phrases.

Final aggregation quantifies the contribution of phrases to the prediction tasks and predictions. One of the authors annotated a list of top 20 phrases per prediction task for each combination of baseline and transformer classifier with different explanation approaches, categorizing them as correctly assigned, incorrectly assigned, or invalid. This enables quantitative comparison of the proposed approaches.

## Results

We present our results in 3 parts. First, we evaluate the predictive performances of the linear bag-of-words and of the transformer models on the outcomes of care need assessments. Note that in this article, this is only an auxiliary result, as we are interested in a good explanation for classification but not optimal classification scores. However, since we want to extract insights from the survey notes using the explanations of the classifiers, their predictive performance is important. Next, we extract insights from these models by obtaining global explanations for each prediction task through aggregating local feature attributions from individual predictions.

### Classification

**Figure 2.**
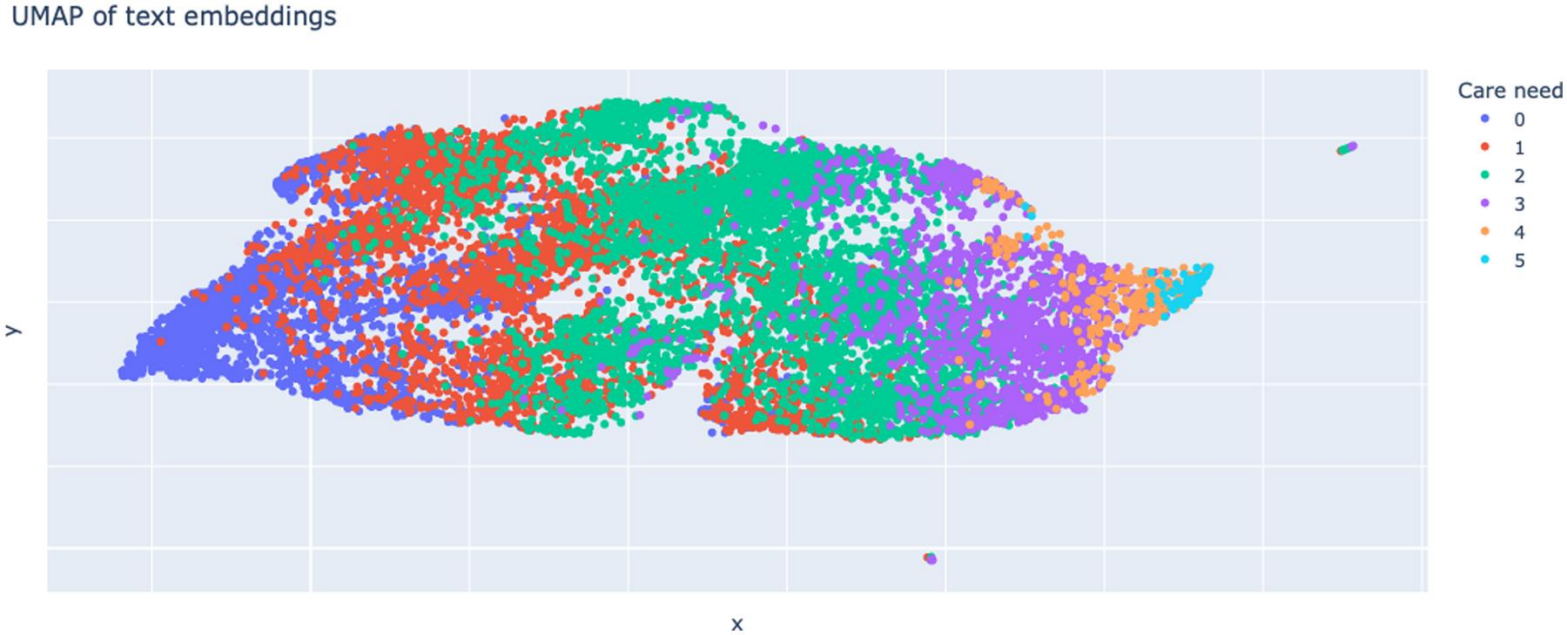
Two-dimensional projection of the embeddings for the assessment texts from the test split with coloring according to the ground truth care need label. Note that the model used to obtain the embeddings have been trained with coarse label set with 3 labels.

The level of care need is the final result of an MD assessment, which is a weighted average from the scores of 6 modules. Together, these modules and the final care need assessment comprise the 7 classification tasks for which we train multi-class classifiers. Table 1 presents these results. We consider 2 settings for the granularity of predictions: The original setting where we have 6 classes for care need task and 5 classes for each module task, and coarse setting (c) considering only 3 classes for each task to make the further analysis with explainability methods easier. The coarser classes for all tasks are obtained using the mapping: 0 → 0; 1,2 → 1; 3,4,5 → 2. We consider 2 classifiers: TF-IDF-based linear bag-of-words model (BoW) and BERT-based transformer model (BERT). As expected, both achieve higher prediction scores for the coarse setting. The transformer model achieves a 4 percentage point improvement in the coarse setting over the BoW model and 7.5 percentage point when predicting all classes. Using default BERT architecture and truncating input to the first 512 tokens (tr) results in significantly worse performance, below that of the BoW model. To further demonstrate the capabilities of the learned model, we visualize the transformer embeddings of the assessment notes from the test set, that is, classifier input, projected onto a 2-dimensional plane using UMAP in Figure 2, which shows an ordered separation indicating that the model trained only on coarse labels effectively captures the ordinality (order) of the full care need classes.

**Table 1.** Evaluation of care-need and module classification tasks with bag-of-words ridge classifier (BoW) and transformers-based BERT (BERT) on truncated texts to BERT’s sequence length (tr) and with architecture modification allowing for full input (pool), using all classes: 6 for care need and 5 for modules, and coarse (c) setting with 3 classes for each task using the mapping 0 → 0; 1,2 → 1; 3,4,5 → 2.

	Outcome	Modules						
	Care need	Mobility	Cog. skills	Behavioral prob.	Self-suff.	Self-therapy	Everyday	Avg
BoW	57.64	69.05	66.29	45.47	65.46	47.51	60.58	58.86
BoW (c)	**79.62**	**82.81**	**82.62**	**66.44**	**79.62**	**64.94**	**76.84**	**76.12**
BERT (tr)	54.97	53.33	54.15	37.63	57.38	41.27	51.72	50.06
BERT	71.74	72.38	68.91	46.74	72.13	62.78	63.09	65.40
BERT (c)	**85.31**	**86.03**	**85.27**	**71.84**	**82.14**	**75.28**	**80.04**	**80.84**

All scores are F1 macro scores over the classes of each task on the test set. Best scores for both the baseline and transformers approach are presented in bold.

### Qualitative exploration of global explanations

The main goal of this work is to discover what contributes to the care need assessment. Feature attribution methods can explain ML model outputs with respect to their inputs. We first explain each prediction task for samples from the training set to derive an attribution score for each task and each token in the input text. Afterwards, we post-process to obtain phrases from tokens and then calculate the global attribution scores for each phrase-task pair across the evaluation set by average (Avg) and homogeneity weighted aggregation (HWA). We use the models from coarse setting as they perform better, and the number of classes complicates our analysis.

Table 2 presents top 5 most attributed phrases identified by average and HW aggregation of explanations from both BoW and transformer models using LRP and IG. We observe that BoW finds more frequent terms, especially when we explain the model by multiplying coefficients with average input, named BoW N. Average aggregation over the dataset delivers rare words for both transformers and baseline BoW model (IG Avg, LRP Avg, Bow Avg). For transformers, average aggregation tends to find mostly single occurrences. When we set a minimum occurrence cutoff, the results did not change, and the top phrases identified by average aggregation had the cutoff frequency. Transformers model demonstrate more sensitivity to rare phrases, successfully identifying meaningful words even if they have typos, such as housekeeping (“hausw(i)rtschaft”). We hypothesize that this may be attributed in part to the pre-training of the transformer model, which is already capable of encoding the complex semantics of words in their context, together with byte pair encoding that incorporates sub-tokens. While average aggregation tends to surface phrases occurring once that are interesting but less useful for overview purposes, HWA captures both rare and common phrases, helping to reduce redundancy of synonymous terms. The distinct phrases obtained by these methods suggest value in using them in combination.

**Table 2.** Top 5 most attributed phrases aggregated using homogeneity weighting (HWA) obtained from transformer model using integrated gradients (IG) and layer-wise relevance propagation (LRP) and from TF-IDF-based linear bag-of-words model (BoW) using its coefficients.

Task Method	Eval C, F, I	Phrases
M2 Cog. skills IG Avg	5, 0, 0	panic attacks (1), topic content (1), short-term memory impairment more complex (1), more complex topic content (1), memory disorders drive deficit (1)
M2 Cog. skills IG HWA	4, 0, 1	patchy (6442), short-term memory patchy (3974), long-term memory patchy (1685), simple facts (1990), facts (4082)
M2 Cog. skills LRP Avg	4, 0, 1	semantic dementia (2), rather short-term memory (1), not able to respond (1), no specific reactions (1), no reaction (1)
M2 Cog. skills LRP HWA	5, 0, 0	patchy (7348), short-term memory patchy (4550), short-term memory (11 317), facts (4653), long-term memory (10 468)
M2 Cog. skills BoW Avg	2, 0, 3	awake coma (12), insuree (1), instruction (1948), insuree hardly any situation (9), icd_version (1)
M2 Cog. Skills BoW HWA	4, 0, 1	patchy (8034), short-term memory preserved (2071), facts (4640), environmental applicant possible (7535), long-term memory preserved (4207)
M2 Cog. Skills Bow N	4, 0, 1	hearing loss (12 082), patchy (9041), instruction (4136), short-term memory patchy (6645), independent (13 315)
M2 Cog. Skills Bow N Hwa	4, 0, 1	patchy (9041), hard of hearing (12 082), facts (7907), short-term memory patchy (6645), situations (6129)
M4 Self-Suff. Ig Avg	2, 3, 0	cooking recipes (1), pronounced delusions (1), therapy sessions (1), various delusions (1), copd suffocation fears (1)
M4 Self-Suff. IG HWA	5, 0, 0	personal hygiene (31 337), clothing (8146), straighten clothing (5377), restricted capping (5097), restricted combing (1000)
M4 Self-Suff. LRP Avg	1, 3, 1	decubiti left (1), memory impairment prompts (1), decubiti left heel (1), various decubiti left (1), left heel buttocks (1)
M4 Self-Suff. LRP HWA	4, 0, 1	body care (37 246), must be cut small (5848), restricted (46 157), restricted fastening (6327), clothing (9169)
M4 Self-Suff. BoW Avg	1, 0, 4	gt independent (52), bds regular (87), both sides regular possible (86), cochlear implant (1), please point (1)
M4 Self-Suff. BoW HWA	3, 0, 2	change of clothing (8681), fastening (9300), clothing (8553), limited independence (6227), centre lower leg possible (8415)
M4 Self-Suff. BoW N	5, 0, 0	change of clothes (11 082), clothes (11 468), closures (11 783), request (11 492), shopping (8816)
M4 Self-Suff. BoW N HWA	3, 2, 0	fastenings (11 783), change of clothes (11 082), mid-lower leg (11 132), mid-lower leg possible (9909), stool incontinence (4103)

For the bag-of-words model, we also present the common approach that uses the average input representation times the linear classifier coefficients. Eval column shows the number of correct, false, and irrelevant phrases as annotated by a human for module allocation and validity. The phrases are provided with their occurrences in parentheses. The original phrases have been translated informally from German and can be found in Table S1.

An important aspect of our work is to separate phrases into different prediction tasks, corresponding to modules of the assessment covering different areas of life. For example, key phrases for the cognitive and communication skills module include phrases such as “strong fears,” “impaired memory & lack of drive,” and “impaired short-term memory,” while the key phrases for the self-therapy module include “Apply compression bandage,” “blood pressure measurement as part of dialysis treatment,” and “abdominal drainage,” for IG, LRP, and BoW, respectively.

Next, we aim to extract risk factors using explanations of cases where the predictive model overestimated the assessment classification compared to the ground truth on the test set. These cases can be seen to lie on the margin of 2 levels, indicating increased risk of wrong assessment outcome. Table 3 shows examples of top-scoring phrases. Here, again our analysis was able to identify significant but rare phrases such as “home respirator,” or “group communication,” which warrants attention. However, it is important to note that while these phrases are mostly associated with predictions of higher care needs, their presence does not necessarily mean an increased care need in all cases.

**Table 3.** Top 5 phrases identified when wrong classifications are explained using LRP and aggregated using homogeneity weighting where the predicted class indicates a higher care need than the actual ground truth class, which can be seen as common risk factors for higher care need.

Task Label ↗ Prediction	Phrases
Care grade 1 ↗ 2	not (1886), annual dialysis (1), palliative care (1), instructions (122), home respirator (3)
M1 Mobility 0 ↗ 2	changing position (2), rehab care bed wheelchair (2), care bed wheelchair rollator (1), care condition care bed lying (1), ventilation therapy at night (1)
M3 Behavioral Prob. 0 ↗ 1	calming (4), occasional anxiety (1), overestimating behavior (1), memory disorders, mood swings (1), takes anxiety (1)
M4 Self-suff. 0 ↗ 1	angry defensive (1), anxiety (1), defensive freedom-depriving (1), hallucinations daily (2), establishing contact angry defensive (1)
M4 Self-Suff. 0 ↗ 2	stair climbing (345), body care (542), spine buttocks area (6), ankle joints (126), calves (16)
M4 Self-Suff. 0 ↗ 2	oral fluid intake (1), oral fluid intake by (1), position changes (2), oral fluid intake prompts (1)
M6 Everyday 0 ↗ 2	significant gaps (1), hallucinations visual ability (1), significant gaps short-term memory (1), visual hallucinations (1), short-term memory visual hallucinations (1)

We explain the predicted class on test data for the simplified setting of 3 classes for all tasks. Prediction column shows the ground truth ↗ prediction. Number of occurrence of a phrase for the same ground truth and prediction pairing is in brackets. The original phrases have been translated informally from German and can be found in Table S2.

### Quantitative evaluation of explanations

**Figure 3.**
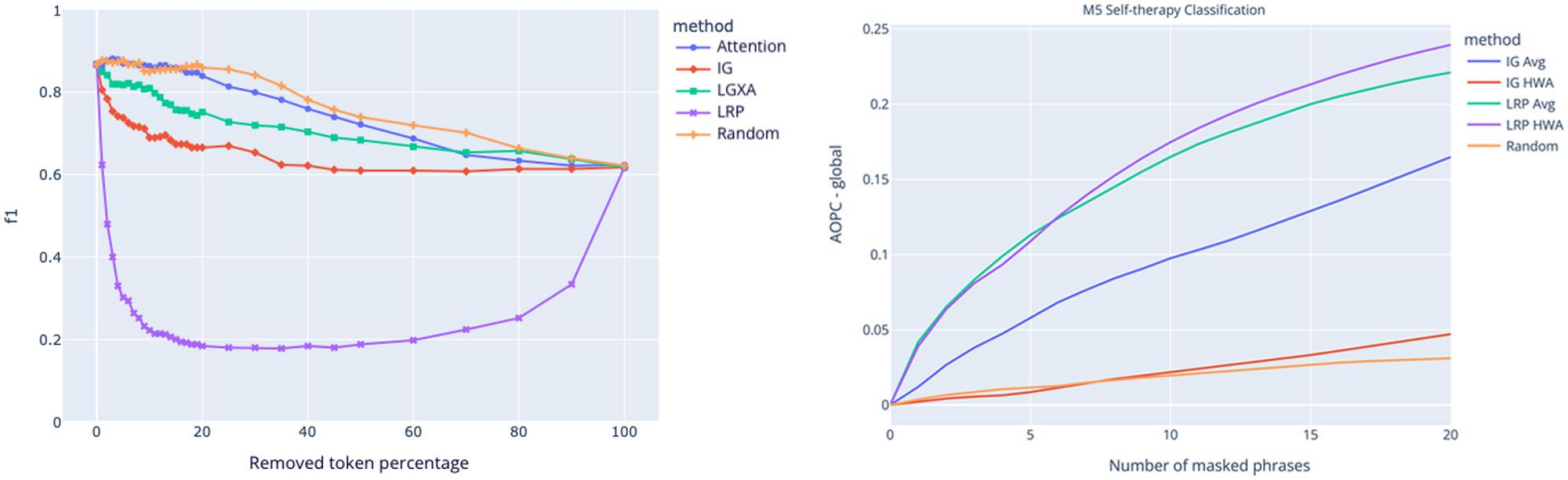
Local and global token removal evaluation: For each sample, we obtain the most attributed tokens using Attention, Layer Integrated Gradients (LIG), Layer gradient × Activation (LGXA), Layer-wise relevance propagation (LRP), and random sampling, and remove tokens incrementally to see their effect on the care need prediction F1 score. Sharper and larger drops in F1 score are better, because it indicates that the attributed tokens are indeed important for the prediction.

We now turn to a quantitative evaluation of the XAI approaches. First, we evaluate the explanations obtained by XAI for the individual predictions of the transformer model. On the left, Figure 3 presents the evaluation of local explanations using token removal,^17^ where we iteratively remove tokens, and repeat the prediction. At each step, F1 score is calculated on the care need prediction task. Sharp drop in the F1 score indicates the identification of phrases that are pertinent to the prediction task. The results show that LRP, distantly followed by IG, identifies by far the most important phrases, which is in line with previous evaluations done on text classifier explanations in the literature.^22^^,^^20^ When we remove all tokens, all the approaches have the same results, close to F1 of 0.35, which corresponds to the prediction accuracy when the model is using the text length as the only information.

On the right, Figure 3 shows evaluation of the globally aggregated explanations using token removal. We use the metric *AOPC_global_* proposed by van der Linden et al.,^21^ for which we iteratively remove tokens using their global attribution rank and calculate the cumulative drop of the prediction logit for the ground truth label, averaged across 500 test samples. Surprisingly, IG attributions with HWA perform similar to random ranking, while for LRP, HWA slightly outperforms average aggregation, consistent with multi-class findings from van der Linden et al.^21^

Table 4 presents the human evaluation for the different models and aggregation methods. To this end, top 20 phrases for each task and method (140 phrases per method) were manually classified as either correct or false assignment to the module task, or irrelevant altogether by one of the authors. Contradictory to the global masked evaluation presented in Figure 3, IG performs similar to LRP and HWA performs slightly better than average aggregation. BoW results show that collecting individual feature attributions do not necessarily improve the quality of phrases. The naive approach to multiply coefficients with the average input representation works well; however, homogeneity aggregation on this naive feature attribution performs substantially better in allocating phrases to correct tasks.

**Table 4.** Human evaluation for the separation of important phrases into different modules.

Method	Correct	False	Irrelevant	Occurrences in	/out of corpus
IG HWA	0.87	0.01	0.13	3383	52
LRP HWA	0.86	0.00	0.14	6067	50
LRP Avg	0.83	0.09	0.08	2	0
IG Avg	0.83	0.13	0.04	1	0
BoW N HWA	0.75	0.02	0.24	6429	100
BoW HWA	0.73	0.03	0.25	5050	47
BoW N	0.64	0.01	0.35	8801	332
BoW Avg	0.58	0.08	0.35	757	12

Top 20 phrases with regard to their aggregated attribution score have been annotated to be correct or false assignment to a particular module or irrelevant altogether. Also we show the average number of occurrence in and out of corpus for the found phrases. As out of corpus, we use German Wikipedia.

We also present the occurrence of top-20 phrases within the corpus and outside the corpus using Wikipedia in Table 4. (We use the same number of documents randomly sampled from https://de.wikipedia.org as we have in the assessment corpus.) Average aggregation delivers phrases occurring mostly once, whereas HWA delivers more common occurring phrases. This makes the average aggregation less useful as the rare phrases it identifies are not necessarily rare occurrences but rare descriptions of common themes, which can be seen as noise. HWA, on the other hand, finds phrases that are rare or common.

## Discussion

Using XAI approaches, we extracted insights deep learning models trained and compared them to interpretable linear models. Global HWA of the individual prediction explanations was key to assigning the insights to different assessment modules. Linear bag-of-words classifiers achieved levels of predictive performance that are approximately 4 percentage points worse than transformer models. Both transformers and bag-of-words models were able to identify rare but important insight phrases. Transformers tended to find more phrases that are rare, whereas bag-of-words tended to find more common phrases. We suggest using different XAI methods together to obtain most comprehensive set of insight phrases.

Our approach leverages multi-task supervision to extract insights through a framework that effectively functions as supervised topic modeling. While related approaches like Supervised Latent Dirichlet Allocation exist,^23^ current implementations lack adaptability to multi-task, multi-label scenarios. Moreover, they are unable to utilize the latest advancements in deep learning, which limits their applicability to our use case. Additionally, although numerous XAI methods are available (eg, LIME, DeepLIFT), given the human-interpretable baseline model, we exclude surrogate explanation techniques like LIME.^24^ Such methods explain predictions of complex machine learning models by building simpler, interpretable models. Similarly, while approaches like DeepLIFT^25^ offer insights into feature importance, they are less suited for transformer-based text models due to architectural incompatibilities. Thus, our approach balances task-specific performance with transparency by design, circumventing the need for external XAI frameworks. Furthermore, we handle long texts with an architectural modification that allows the use of state-of-the-art German clinical pretrained transformer, instead of utilizing a model that allows longer input, such as LongFormer, but are no German clinical pretrained weights available for these models.

Global task importance aggregation can be used to assess the completeness of assessment instruments. By applying homogeneity weighting, we can identify phrases especially important for care need classification highlighting phrases important for overall score prediction but not included in the assessment modules, thereby revealing gaps in the instrument. Our examination of top phrases assigned to care need did not reveal any such gaps; the phrases deemed important for care need are adequately covered by existing assessment modules. Most phrases were correctly assigned to their respective assessment modules.

A general of challenge of research in XAI methods is evaluating and interpreting their results. To address this, we conducted both quantitative and qualitative assessments, including human annotation. Using both revealed a notable discrepancy: LRP outperformed IG in the quantitative evaluation of local explanations using token removal, yet it underperformed in the aggregate-level human evaluation. This inconsistency highlights the complexity of assessing XAI methods comprehensively. Moreover, the list of top phrases includes many semantically similar expressions with varied formulations. Clustering, as implemented by Dürlich et al,^26^ offers a way to manage this complexity.

Our objective was to obtain insights from the large text notes corpora and the associated structured assessment survey results. The results of the care need assessment has significant impact on both the assessed individual and the society at large because of how societal resources get allocated. We do not see the automation of this process by machine learning as a goal. Rather, we believe that it is important to gain insights from the past assessments and identify patterns and trends from the vast notes generated by the assessments carefully conducted by humans.

However, the practical applicability of these results is constrained by several factors. First, the feature attribution scores derived from these methods prove inadequate for comparative analysis due to their arbitrariness across different methods. Second, the analysis process necessitates a laborious examination of phrases that are removed from their contexts. However, a manual review of the reports would constitute a much laborious examination, and simple solutions to user experience, for example, showing contexts where the phrases are coming from, could improve the practicability of the results.

## Conclusion

In this work, we have explored how to utilize traditional bag- of-words and state-of-the-art deep learning based transformer models to extract insights regarding care need assessment from the accompanying text notes. We performed a human evaluation of the quality of insights obtained through different feature attribution methods and explanation aggregation methods for both transformer-based models and traditional bag-of-words models. The objective was to gain insights into the assessment process and identify key factors influencing the care need of individuals.

We demonstrated the potential of applying deep learning combined with explainable AI techniques to analyze a large corpus of care need assessments. Bag-of-words model proved to still be a useful tool for insight extraction, however only when used in tandem with global aggregation of individual feature attributions. While this work focused on technical aspects of prediction and interpretation, future work could explore how these insights can be effectively integrated into the decision-making processes of the MD.

## Supplementary Material

ooaf064_Supplementary_Data

## Data Availability

The data used for our experiments consist of care need assessment surveys that potentially contain personally identifiable information and health information; as such, we are not authorized to make our dataset publicly available. The aggregate, extracted information resulting from our experiments may be accessible upon request and with subsequent authorized approvals of Medical Service (Medizinische Dienst).
